# Prognostic similarity between ovarian mucinous carcinoma with expansile invasion and ovarian mucinous borderline tumor: A retrospective analysis

**DOI:** 10.1097/MD.0000000000026895

**Published:** 2021-08-13

**Authors:** Taira Hada, Morikazu Miyamoto, Hiroki Ishibashi, Hiroko Matsuura, Takahiro Sakamoto, Soichiro Kakimoto, Hideki Iwahashi, Rie Suzuki, Kimiya Sato, Hitoshi Tsuda, Masashi Takano

**Affiliations:** a Department of Obstetrics and Gynecology, National Defense Medical College Hospital, Saitama, Tokorozawa, Japan; b Department of Pathology, National Defense Medical College Hospital, Saitama, Tokorozawa, Japan.

**Keywords:** expansile invasion, ovarian mucinous borderline tumor, ovarian mucinous carcinoma

## Abstract

There is a similarity of histological features and survival between ovarian mucinous carcinoma (MC) with expansile invasion and ovarian mucinous borderline tumor (MBT). The aim of this study was to compare the clinical outcomes of MC with expansile invasion with those of MBT based on the 2020 World Health Organization (WHO) criteria.

A pathological review was performed on patients with MC, ovarian MBT, and seromucinous borderline tumors that underwent surgery at our hospital between 1984 and 2019. Clinicopathological features were compared retrospectively between MC with expansile invasion and MBT.

Among 83 cases of MC, 85 cases of MBT, and 12 cases of seromucinous borderline tumor, 25 MC cases with expansile invasion and 98 MBT cases were included through review. MC cases with expansile invasion were diagnosed with advanced International Federation of Gynecology and Obstetrics (FIGO) stages more frequently (*P* = .02) than that of MBT cases. In addition, patients with MC with expansile invasion received adjuvant chemotherapy more often (*P* < .01) than that of patients with MBT. There were no statistically significant differences in recurrence rate (*P* = .10) between MC with expansile invasion and MBT. Progression-free survival (PFS) was worse in MC cases with expansile invasion than that in MBT cases (*P* = .01). However, a multivariate analysis for PFS showed that histological subtype, FIGO stage, and adjuvant chemotherapy were not an independent prognostic factor.

The prognostic outcome of MC with expansile invasion might mimic those of MBT. These results showed ovarian borderline tumor treatment could be applied to MC treatment.

## 1 Introduction

Ovarian carcinoma (OC) is the seventh most common cancer in women and has been increasing recently.^[1]^ Histological subtypes have been recognized as important prognostic factors. Among the subtypes, the incidence of mucinous carcinoma (MC) ranges from 3% to 11% in Western countries and over 10% in most Asian countries, with its frequency varying among each region.^[2,3]^ Furthermore, MC has been detected in early stages of cancer, has a lower response to chemotherapy, and has been associated with a worse prognosis, particularly in advanced stages than in high-grade serous carcinomas.^[2,4–6]^ Due to the relatively low incidence of MC, appropriate individual treatment has not been established.

The 2020 World Health Organization (WHO) classification notes that 2 invasive patterns, infiltrative invasion and expansile invasion, are associated with prognosis.^[7]^ Infiltrative patterns are associated with a worse prognosis; hence, expansile patterns are associated with a better prognosis, and the same associations are true in cases diagnosed as International Federation of Gynecology and Obstetrics (FIGO) stage I.^[8–11]^ Among MC cases with infiltrative invasion, 40.0% to 54.5% cases are diagnosed as FIGO stage II to IV, and the recurrence rate ranges from 54.5% to 75.0%.^[8–10]^ Conversely, among MC cases with expansile invasion, 91.3% to 100% are diagnosed as FIGO stage I, and the recurrence rate ranges from 0% to 9.5%.^[8–10]^ The 2019 European Society for Medical Oncology and European Society of Gynaecological Oncology consensus conference recommendations on ovarian cancer initially implemented the invasive pattern as the factor to make a decision to perform adjuvant therapy, and specifically recommended to omit or optionally choose adjuvant chemotherapy for MC with expansile invasion at FIGO stage I.^[12]^ Therefore, an invasive pattern in MC has been recognized as an important prognostic factor.

In contrast to OCs, ovarian borderline tumors (OBTs) are neoplasms with higher epithelial proliferation and variable nuclear atypia without destructive stromal invasion.^[7,13]^ OBTs are often diagnosed at an earlier stage and in younger women. They have a better prognosis than OCs, with a 97% ten-year survival for all stages.^[13–15]^ The recurrence rate ranges from 3% to 10%, and 32% of recurrence occur after more than 5 years after diagnosis.^[16–18]^ As postoperative management, unlike OCs, adjuvant chemotherapy is not recommended for cases without implants but is an optional choice for cases with implants.^[18–20]^ The 2020 WHO criteria classified mucinous borderline tumors as either mucinous borderline tumor (MBT), defined as a gastrointestinal-type, or seromucinous borderline tumor, defined as an endocervical type.^[7]^ Among OBTs, the incidence of MBT ranges from 26.7% to 50%, and they have a good prognosis with a 10-year survival rate of 94.0%.^[7,13,15,21–23]^

There is a similarity in not only the degree of nuclear atypia but also the survival when confined to the ovary at presentation between MC with expansile invasion and MBT.^[13]^ However, there are no reports comparing MC with expansile invasion with MBT. Herein, the aim of this study was to identify MC cases with expansile invasion and MBT through a pathological review and evaluate the clinicopathologic and prognostic characteristics of MC with expansile invasion and MBT.

## 2 Methods

**Figure 1:**
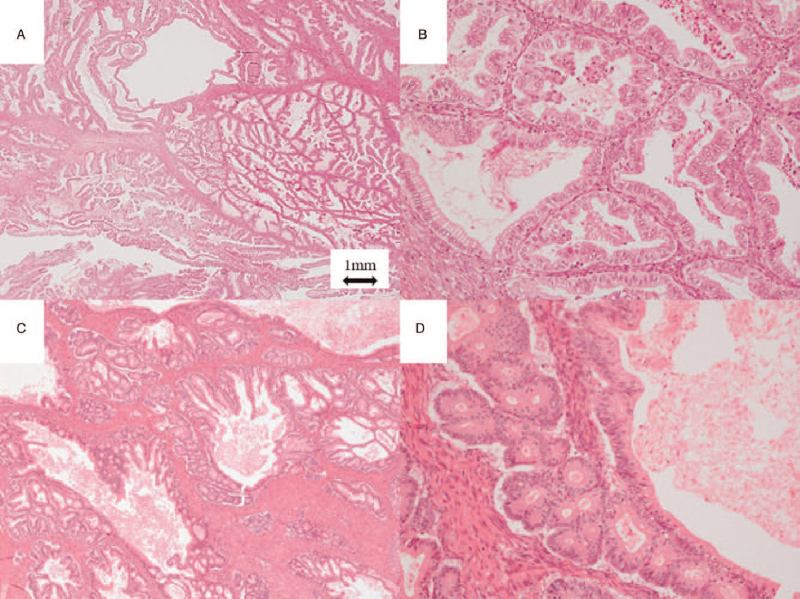
Representative images of mucinous carcinoma (MC) with expansile invasion and mucinous borderline tumor (MBT). (A) MC with expansile invasion was marked as glandular crowding, creating a labyrinthine appearance with little intervening normal ovarian stroma (×20). (B) Back to back malignant glands were seen in MC cases with expansile invasion (×200). (C) MBT demonstrated cystic glandular structures with papillary infoldings, columnar cells with abundant cytoplasmic mucin, and were admixed with goblet cells of variable degrees of maturation (×20). (D) Basally located nuclei with no considerable nuclear atypia were seen in MBT (×200).

Our analysis included patients with MC, ovarian MBT, and seromucinous tumors who underwent surgery at our hospital between 1984 and 2019. The exclusion criteria included cases without clinical information, surgical tissue, and a history of chemotherapy or other carcinomas. A pathological review was performed based on morphology alone using the 2020 WHO criteria by 2 pathologists who performed a part of original pathological diagnoses without clinical information.^[7]^ Patients with MC with expansile invasion and MBT were included in our analysis. Briefly, the definition of MC with expansile invasion was a malignant epithelial tumor composed of gastrointestinal-type cells containing intra-cytoplasmic mucin accompanied by an expansile pattern recognized by marked glandular crowding with little intervening stroma and creating a labyrinthine appearance. The definition of MBT was a tumor composed of mild to moderately atypical gastrointestinal-type and mucin-containing epithelial cells with or without microinvasion, defined as a microinvasion measuring less than 5 mm in greatest linear extent. We used the calibration of at least x200 to estimate the nuclear atypia. Representative images of MC with expansile invasion and MBT are shown in Figure 1.

Clinical information was collected from patient medical records. Staging was evaluated using the 2014 FIGO criteria.^[24]^ Residual tumors were obtained from the operation records of the primary surgeries. The Response Evaluation Criteria in Solid Tumors version 1.1 was used to evaluate the efficacy of chemotherapy.^[25]^ Progression-free survival (PFS) was defined as the period from the day of primary surgery to the day of death or recurrence/progression of the disease. Overall survival (OS) was defined as the period from the day of primary surgery to the day of death or last confirmation of their existence. This study included many cases in our previous reports^[26,27]^ We added newly several cases and conducted prognostic analysis with extended follow-up period.

Statistical analyzes were performed using JMP Pro 14 software (SAS Institute Inc., Cary, NC, USA). The Chi-Squared test, Fisher exact test, and Mann–Whitney *U* test were used to evaluate the clinical significance of clinicopathological factors. PFS and OS curves were generated using the Kaplan–Meier method. The log-rank test was used to compared survival distributions. A Cox proportional hazard was used for multivariate analysis of PFS. Statistical significance was defined as a *P* value <.05.

This study was approved by the ethical committee of our hospital at the National Defense Medical College, Tokorozawa, Japan (approval number: 4130). Because our study was retrospective analysis, informed consent did not be obtained. However, the chance of refusal to participate out study was put on the website of our hospital and we ensure its chance.

## 3 Result

**Figure 2:**
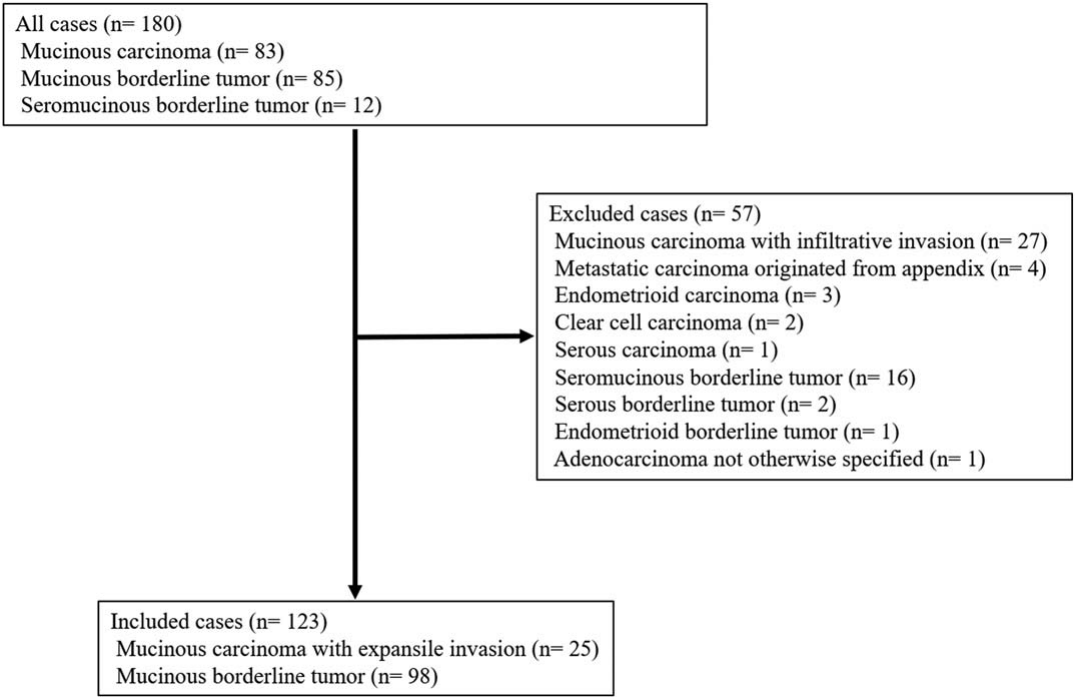
Changes of histological types of all cases through pathological review. Eighty 3 cases with mucinous carcinoma (MC) and 85 cases with mucinous borderline tumor (MBT) were identified. Ultimately, 25 patients with MC with expansile invasion and 97 patients with MBT were included.

Using initial pathological diagnosis, our study identified 180 cases: 83 cases of MC, 85 cases of ovarian MBT, and 12 cases of seromucinous borderline tumor. The median number of blocks of ovarian tumor was 10 (2–35). The results of the pathological review are shown in Figure 2. Among 83 MC cases, 25 were diagnosed as MC with expansile invasion, 17 as MBT, 27 as MC with infiltrative invasion, 4 as metastatic carcinoma originating from the appendix, 3 as endometrioid carcinoma, 2 as clear cell carcinoma, one as serous carcinoma, 3 as seromucinous borderline tumors, and one as adenocarcinoma not otherwise specified through review. In addition, among the 85 cases with MBT, 80 cases were diagnosed with MBT, and 5 cases with seromucinous borderline tumor. Moreover, among the 12 cases with seromucinous borderline tumors, 1 case was diagnosed with MBT, 8 with seromucinous borderline tumor, 2 with serous borderline tumor, and 1 with endometrioid borderline tumor. Ultimately, our study included 123 cases: 25 cases of MC with expansile invasion and 98 cases of MBT.

**Figure 3:**
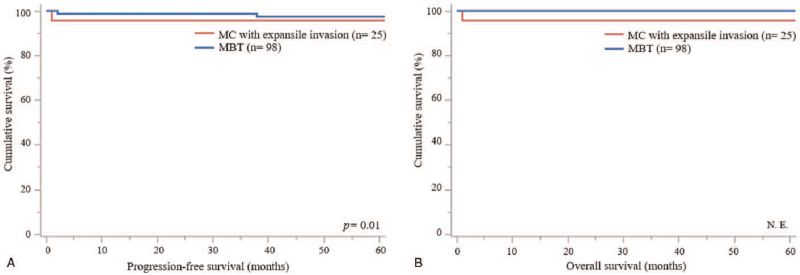
Progression-free survival (PFS) curves and overall survival (OS) curves of mucinous carcinoma (MC) cases with expansile invasion with and without chemotherapy and mucinous borderline tumor (MBT). (A) MC cases with expansile invasion had a worse PFS than that of MBT cases (*P* = .01). (B) OS curves of the MC cases with expansile invasion and MBT (Not evaluable: N.E.).

The median follow-up period was 61 months (range, 1–305 months). The characteristics of MC cases with expansile invasion and MBT cases are shown in Table 1. Patients with MC with expansile invasion were diagnosed with more advanced stages (*P* = .02) and received adjuvant chemotherapy (*P* < .01) more frequently than that of patients with MBT. The mean tumor size of MC with expansile invasion and MBT was 20.1 centimeters and 18.3 centimeters, respectively (*P* = .89). There were no statistically significant differences in other factors between MC with expansile invasion and MBT. MC cases with expansile invasion had a worse PFS than that of MBT cases (Fig. 3a; *P* = .01), and OS could not be evaluated because all patients with MBT were alive (Fig. 3b; not evaluable). Univariate analysis for PFS revealed that histological type (hazard ratio 6.15; *P* = .03) was identified as a prognostic factor, however, multivariate analysis for PFS demonstrated that histological subtype was not a prognostic factor (hazard ratio 3.49; *P* = .15) (Table 2).

**Table 1:** Characteristics of ovarian mucinous carcinomas with expansile invasion and mucinous borderline tumor/atypical proliferative mucinous tumors.

	MC with expansile invasion	Mucinous borderline tumor	
Variables	n = 25	n = 98	*P* value
Age (yr) (n (%))			.38
<51	14 (56.0)	45 (45.9)	
≥51	11 (44.0)	53 (54.1)	
FIGO stage (n %))			.02
I	20 (80.0)	94 (95.9)	
II	2 (8.0)	1 (1.0)	
III	2 (8.0)	3 (3.1)	
IV	1 (4.0)	0 (0)	
FIGO stage I subclassification (n (%))			.01
IA	8 (40.0)	66 (70.2)	
IB	0 (0.0)	0 (0.0)	
IC1	6 (30.0)	7 (7.4)	
IC2	0 (0.0)	4 (4.3)	
IC3	6 (30.0)	17 (18.1)	
Tumor size (cm)			.89
Mean ± Standard Deviation	20.1 ± 11.9	18.2 ± 7.6	
Peritoneal cytology (n (%))			.07
Positive	10 (40.0)	20 (20.4)	
Negative	15 (60.0)	78 (79.6)	
Residual tumor at primary surgery (n (%))			.10
Positive	3 (12.0)	3 (3.1)	
Negative	22 (88.0)	95 (96.9)	
Adjuvant chemotherapy (n (%))			<.01
Not done	12 (48.0)	84 (85.7)	
Done	13 (52.0)	14 (14.3)	
Response rate of adjuvant chemotherapy for cases with macroscopic residual tumor at primary surgery (n (%)) ^∗^			.40
CR/PR	1 (50.0)	3 (100)	
SD/PD	1 (50.0)	0 (0.0)	
Recurrence (n (%))			.10
Yes	2 (8.0)	1 (1.0)	
No	23 (92.0)	97 (99.0)	

**Table 2:** Univariate and multivariate analyzes of progression-free survival in all cases.

	Univariate analysis		Multivariate analysis	
Variables	Hazard ratio (95% confidence interval)	*P* value	Hazard ratio (95% confidence interval)	*P* value
Age (yr)				
≥ 51 vs <51	2.22 (0.43–15.95)	.35		
FIGO stage				
I, II vs III, IV	0.13 (0.03–0.98)	.048	0.33 (0.06–2.49)	.25
Residual tumor at primary surgery				
Positive vs negative	6.25 (0.85–33.04)	.07		
Peritoneal cytology				
Positive vs negative	1.05 (0.14–5.45)	.96		
Adjuvant chemotherapy				
Done vs not done	11.52 (1.80–223.28)	<.01	5.72 (0.72–120.31)	.10
Histological type				
MC with expansile invasion vs. MBT	6.15 (1.19–44.73)	.03	3.49 (0.65–24.40)	.15

The characteristics of 2 cases with MC with expansile invasion and 1 case with MBT that recurred are shown in Table 3. In the first case of MC with expansile invasion, a 60-years-old woman underwent right salpingo-oophorectomy, peritoneal biopsy, and appendectomy. Her diseases were diagnosed as FIGO stage IIIC and she did not receive adjuvant chemotherapy because of her demand. Her cancerous pleurisy recurred at 91 months after the day of primary surgery. She did not receive treatment for recurrence because of her poor condition and personal choice. She died of the disease 4 months after the day of recurrence. In the second case of MC with expansile invasion, a 69-years-old woman underwent right salpingo-oophorectomy, omentectomy, and pelvic and para-aortic lymphadenectomies followed by chemotherapy, including a combination of etoposide and cisplatin. Her diseases were diagnosed as FIGO stage IC3 and recurred 91 months after the day of primary surgery. Combination chemotherapy, consisting of irinotecan and nedaplatin, was administrated but the patient did not respond, and she died 17 months after the recurrence.

**Table 3:** Details of three cases with recurrence.

Histological type	Age (Years)	FIGO stage	Surgical form of primary surgery	Residual tumor (cm)	Peritoneal cytology	Adjuvant chemotherapy	PFS (Months)	Site of recurrence	Treatment after recurrence	OS (Months)	Status
MC with expansile invasion	60	IIIC	RSO + peritoneal biopsy +appendectomy	2	Positive	–	91	Cancerous pleurisy	Not done	95	Died of disease
MC with expansile invasion	69	IC3	RSO + omentectomy + pelvic and para-aortic lymphadenectomy	0	Positive	Etoposide + cisplatin	91	Vaginal stump	Chemotherapy (irinotecan + nedaplatin)	108	Died of other cause
MBT	53	IA	RSO + omentectomy	0	Negative	Etoposide + cisplatin	38	Vaginal stump	Surgery	62	No evidence of disease

The first case of MBT was in a 53-year-old woman who underwent right salpingo-oophorectomy and omentectomy as an initial first treatment followed by chemotherapy. Her diseases were diagnosed as FIGO stage IA and recurred at the vaginal stump 38 months after the first treatment. Her tumor was resected. She was alive 62 months after the first treatment and she was transferred to another hospital.

## 4 Discussion

In our study, 25 patients with MC with expansile invasion and 98 patients with MBT were analyzed. More patients with MC with expansile invasion were diagnosed with advanced stage diseases and received adjuvant chemotherapy more often than that of patients with MBT. MC with expansile invasion had worse PFS than that of MBT. Furthermore, univariate analysis revealed that FIGO stage, adjuvant chemotherapy, and histological type were related to PFS, but multivariate analysis showed that these factors were not prognostic factors for PFS.

The diagnosis of mucinous tumors has been given increased attention, as the 2020 WHO criteria has modified the diagnosis of mucinous tumors, and previous reports are in disagreement with the new modifications. Specifically, the 2020 WHO criteria defined a microinvasion as less than 5 mm in greatest linear extent^[7]^; however, several previous reports cited the definition of a microinvasion as 10 mm^2^ in area, which was calculated to be over 3 mm in each of the 2 liner dimensions.^[8,9,28]^ A seromucinous borderline tumor has been newly defined. The difficulty of the diagnosis is that since benign tumors, borderline tumors, and invasive carcinomas can co-exist, and it can be easy to miss MC.^[29–32]^ Furthermore, appropriate sampling of tissue is needed to diagnose foci of invasion because mucinous tumors are large.^[33]^ In our study, multiple cases (20.6%) were needed to change the diagnosis after pathological review in order to apply the 2020 WHO criteria. Research on mucinous tumors requires an exact pathological re-evaluation. In addition, further research is needed to examine the method to make the sample. Furthermore, many cases were changed their diagnosis through review, which might indicate that the diagnostic criteria for MC and MBT might be insufficient. The new diagnostic criteria for MC and MBT which also referred about magnification would be desirable to be made in the future.

Previous studies have investigated the effectiveness of adjuvant chemotherapy for early stage OCs. According to the International Collaborative Ovarian Neoplasm Trial 1 and Adjuvant Chemo Therapy in Ovarian Neoplasm trial, platinum-based adjuvant chemotherapy improved the survival of patients with early stage OC.^[34,35]^ However, other retrospective analyses limited to MC showed no benefit from adjuvant chemotherapy.^[36,37]^ Furthermore, the effectiveness of adjuvant chemotherapy for MBT has not been demonstrated.^[38,39]^ In our study, adjuvant chemotherapy was not a prognostic factor. Therefore, our results might indicate patients with MC with expansile pattern may not need adjuvant chemotherapy as MBT.

According to the recommendation of ESMO guidelines, the follow-up period after the primary treatment for 5 years for OC was every 3 to 4 months for the first 2 years and every 6 months for 3 to 5 years. An additional follow-up beyond 5 years is individually decided.^[12]^ Additionally, according to the recommendation of the 2018 National Comprehensive Cancer Network guidelines, the follow-up period after the primary treatment for OBT was every 3 to 6 months for 5 years, and that for OC was every 2 to 4 months for the first 2 years and 3 to 6 months during 3 to 5 years with long-term wellness care extending beyond 5 years being recommended for both OBT and OC.^[40]^ In our study, the duration from primary treatment to recurrence in 2 recurrent MC cases with expansile invasion was longer in addition to exhibiting a low recurrence rate similar to that of MBT cases. Thus, our results might show the possibility that follow-up intervals and periods could be extended.

The Gynecologic Oncology Group trial 241 compared treatments of either carboplatin-paclitaxel with or without bevacizumab or oxaliplatin-capecitabine with and without bevacizumab and showed no survival benefits from these treatments.^[41]^ A previous report demonstrated no benefits from the combination of oxaliplatin and 5-fluorouracil, which is traditionally used for gastrointestinal cancers.^[42]^ Therefore, an effective regimen has not been established until now. Our study demonstrates the possibility that MC with expansile invasion may not be effectively treated by adjuvant chemotherapy, has a low recurrence rate, and has a long duration until recurrence. However, 2 recurrent MC cases with expansile invasion had no benefit from chemotherapy at recurrence and died of diseases relatively promptly after recurrence. Therefore, a new treatment for MC with expansile invasion at recurrence is needed, particularly at recurrence.

According to a previous review that included MC cases with both invasive patterns, a KRAS mutation occurred in 33% to 46% of cases, TP53 mutation in 26% to 55%, and HER-2 mutation in 18% to 35% of cases.^[43]^ Additional reports also showed that amplification and overexpression of HER-2 was observed in MC with expansile invasion, and trastuzumab therapy was proposed as a treatment option for MC cases with amplification and overexpression of HER-2.^[44,45]^ Because several reports demonstrated that the biological and clinical behaviors of both invasive patterns were clearly different, further studies are needed to develop new treatments including an investigation of the genetic background of each invasive pattern.

The present study has several limitations that should be considered. Our analysis included a small sample size at a single institution and was conducted retrospectively. Further studies with a larger sample size are required to confirm the clinical significance of MC with expansile invasion and MBT in the future. In addition, the problem of the difficulty in diagnosis due to the large tumor size should be addressed. Moreover, in our hospital, the method of making sample sections and the original diagnosis of mucinous ovarian tumors were conducted based on the WHO classification after 2003 WHO classification mentioned the numbers of sample sections and mural nodules in mucinous tumors. Because, before 2003, there were no definitive protocol to make samples and diagnosis sample sections of mucinous tumors might be insufficient and mural nodules might not be sufficiently observed. However, although there are some restrictions, we believed our results might be useful for clinical settings because the pathological review was carefully performed.

In conclusion, through a clinicopathological review of MC cases with expansile invasion and MBT, MC with expansile invasion had similar clinical features as MBT excluding that MC with expansile invasion were diagnosed at more advanced stage, and there were no statistical differences in prognosis between these groups. Our results indicate that the same management principles for OBT cases could potentially be applied to MC cases with expansile invasion.

## Acknowledgments

We would like to thank Editage for help with language editing of the manuscript.

## Author contributions

**Conceptualization:** Taira Hada, Morikazu Miyamoto, Masashi Takano.

**Data curation:** Taira Hada, Morikazu Miyamoto, Masashi Takano.

**Formal analysis:** Taira Hada, Morikazu Miyamoto, Kimiya Sato, Hitoshi Tsuda.

**Investigation:** Taira Hada, Soichiro Kakimoto, Hideki Iwahashi.

**Methodology:** Taira Hada, Hiroki Ishibashi, Hiroko Matsuura, Takahiro Sakamoto.

**Project administration:** Taira Hada.

**Resources:** Hiroki Ishibashi.

**Supervision:** Rie Suzuki.

**Validation:** Morikazu Miyamoto.

**Visualization:** Morikazu Miyamoto.

**Writing – original draft:** Taira Hada.

**Writing – review & editing:** Morikazu Miyamoto, Masashi Takano.
